# Carrion Beetles Visiting Pig Carcasses during Early Spring in Urban, Forest and Agricultural Biotopes of Western Europe

**DOI:** 10.1673/031.011.7301

**Published:** 2011-06-29

**Authors:** Jessica Dekeirsschieter, François J. Verheggen, Eric Haubruge, Yves Brostaux

**Affiliations:** ^1^ Department of Functional and Evolutionary Entomology, Gembloux Agro-Bio Tech, University of Liege, 2 Passage des Déportés, 5030 Gembloux, Belgium; ^2^ Department of Applied Statistics, Computer Science and Mathematics, Gembloux Agro-Bio Tech, University of Liege, 2 Passage des Déportés, 5030 Gembloux, Belgium

**Keywords:** Silphidae, carrion ecology, decomposition, forensic entomology, insect succession

## Abstract

Carrion beetles are important in terrestrial ecosystems, consuming dead mammals and promoting the recycling of organic matter into ecosystems. Most forensic studies are focused on succession of Diptera while neglecting Coleoptera. So far, little information is available on carrion beetles postmortem colonization and decomposition process in temperate biogeoclimatic countries. These beetles are however part of the entomofaunal colonization of a dead body. Forensic entomologists need databases concerning the distribution, ecology and phenology of necrophagous insects, including silphids. Forensic entomology uses pig carcasses to surrogate human decomposition and to investigate entomofaunal succession. However, few studies have been conducted in Europe on large carcasses. The work reported here monitored the presence of the carrion beetles (Coleoptera: Silphidae) on decaying pig carcasses in three selected biotopes (forest, crop field, urban site) at the beginning of spring. Seven species of Silphidae were recorded: *Nicrophorus humator* (Gleditsch), *Nicrophorus vespillo* (L.), *Nicrophorus vespilloides* (Herbst), *Necrodes littoralis* L., *Oiceoptoma thoracica* L., *Thanatophilus sinuatus* (Fabricius), *Thanatophilus rugosus* (L.). All of these species were caught in the forest biotope, and all but *O. thoracica* were caught in the agricultural biotope. No silphids were caught in the urban site.

## Introduction

Carrion beetles (Coleoptera: Silphidae) perform vital ecosystem functions (Wolf and Gibbs 2004) by promoting the breakdown and recycling of organic matter into terrestrial ecosystems (Ratcliffe 1996; Hastir and Gaspar 2001; Kalinova et al. 2009). Most silphids are carrion feeders (necrophagous species) or prey on other carrion inhabitants such as fly eggs or maggots and other carrion beetles (necrophilous species) (Ratcliffe 1996; Hastir and Gaspar 2001; Sikes 2005, 2008). The necrophagous insects, including flies and carrion beetles, have particular relationships with decomposing remains of vertebrate carcasses that constitute a rich, but ephemeral resource (Anderson and VanLaerhoven 1996; Grassberger and Frank 2004; Carter et al. 2007). These specialized insects are attracted to the cadaver that they colonize in a relative predictable sequence called the entomofaunal succession or insect succession (Megnin 1894; Putman 1983; Schoenly and Reid 1987; Marchenko 1988, 2001; Benecke 2004). Study of these insects in a medico-legal context is part of forensic entomology (Hall 1990; Amendt et al. 2004). Many forensic entomological studies have been conducted on pig carcasses as surrogate human models for physiological, ethical and economical reasons (Rodriguez and Bass 1983; Catts and Goff 1992; Anderson and VanLaerhoven 1996; Grassberger and Frank 2004; Hart and Whitaker 2005), but few were conducted in Europe with pig carcasses (Grassberger and Frank 2004; Garcia-Rojo 2004; Wyss and Cherix 2006; Matuszewski et al. 2008). Many published reports are focused on Diptera pattern colonization and very few looked at Coleoptera succession (Kocarek 2003; Matuszewski et al. 2008; Midgley and Villet 2009, Midgley et al. 2010). However, the use of beetles in forensic entomology can be relevant (Kulshrestha and Satpathy 2001; Midgley et al. 2010). Families of beetles of forensic importance are Silphidae (carrion beetles), Dermestidae (larder, skin or hide beetles), Staphylinidae (rove beetles), Histeridae (clown or hister beetles), Cleridae (checker beetles) and Nitidulidae (sap beetles) (Haskell et al. 1997; Byrd and Castner 2001; Wyss and Cherix 2006). Among them, carrion beetles can provide information on postmortem colonization on remains and time since death (Haskell et al. 1997; Smith 1986; Watson and Carlton 2005). So far, little information is available on carrion beetles postmortem colonization and the process of decomposition in temperate biogeoclimatic countries.

The world fauna of Silphidae is composed of fewer than 200 species distributed in 15 genera (Portevin 1926; Peck 2001; Sikes 2005). This family has a Holarctic distribution (Peck 2001). In Western Europe, this family is divided in two subfamilies: Nicrophorinae (i.e. burying beetles) with eleven species and Silphinae including seventeen species (Portevin 1926; Du Chatenet 1986; Hastir and Gaspar 2001; Debreuil 2003a,b, 2004a,b,c). There are seven species of Nicrophorinae and thirteen species of Silphinae reported in Belgium (Hastir and Gaspar 2001; Ružicka and Schneider 2004). Many forensic entomological papers highlight the necessity to generate data on insect succession and insect seasonal activity on carrion in specific geographic regions and various biotopes within these regions (Catts and Goff 1992; Byrd and Castner 2001; Amendt et al. 2004; Sharanowski et al. 2008). This paper identifies the early activity of silphids that occur on large carcasses in a temperate biogeoclimatic region in three different biotopes (forest, agricultural and urban site).

## Materials and Methods

### Sites and study period

This study was conducted during spring 2007 (29 March – 11 May) in three distinct biotopes: a forest biotope, an agricultural biotope and an urban site, located in Belgium. The forest habitat consisted of pedonculate oaks, *Quercus robur* L. (Fagales: Fagaceae), European beeches, *Fagus sylvatica* L. and sycamore maples, *Acer pseudoplatanus* L. (Sapindales: Sapindaceae). The agricultural biotope was a transect (5 meters width) of meadow with an alignment of willows (*Salix sp.*) between a barley *Hordeum vulgare* L. (Poales: Poaceae) field and an enclosed grassland. The meadow was not grazed for the duration of the experiment. The urban biotope was an abandoned building of two floors with broken windows and inside vegetation *Clematis vitalba* L. (Ranunculales: Ranunculaceae). The building was located on a secure site belonging to the National Institute of Criminalistic and Criminology (INCC-NICC, Brussels, Belgium).

### Animal model

Six piglets, *Sus domesticus* L. (Artiodactyla: Suidae), (25 Kg) were killed by penetrative captive bolt (fractured skull) and disposed in the experimental sites within the next 4 hours. Immediately after the euthanasia, the pig carcasses were packed in double plastic bag to avoid any insect colonization before being placed in the experimental biotope. In each site, two pig carcasses were placed 50 meters from each other, in metal mesh cages (180 cm × 90 cm × 90 cm) to avoid scavenging by vertebrate carnivores.

### Insect collection and identification

**Figure 1.**
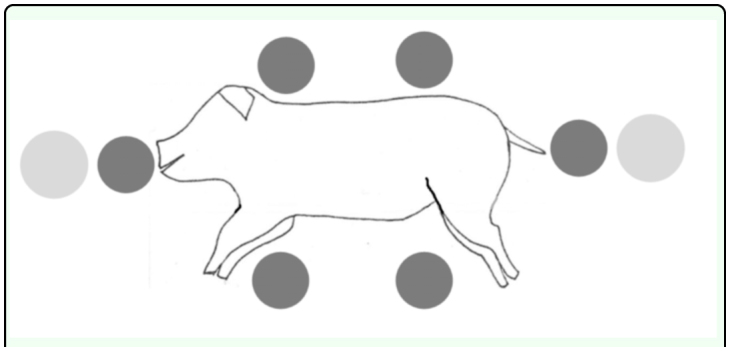
Disposition of the traps around the pig carcass (dark grey, pitfall traps; light grey, yellow traps). High quality figures are available online

In order to quantify insect colonization on pig carcasses, pitfall traps and yellow traps were used to collect sarcosaprophagous insects. For continuous surveillance, six pitfall traps (glass jars of 15 cm in height and 8 cm in diameter) and two yellow traps (plastic container of 9 cm in height and 27 cm in diameter), both filled with soapy water, were placed around each carcass. The disposition of the pitfall traps flush to the surface, was the following: two near the ventral face, two near the dorsal face, one near the head and one near the anus. One yellow trap was placed near the head and the other was placed near the anus (Figure 1).

The insect traps were removed every four days and the collected specimens were conserved in 80% *norvanol D* (ethanol denatured with ether). Only the adult stages were included in the counting of collected insects during this study.

Silphidae specimens were determining using different identification keys (Hastir and Gaspar 2001; Debreuil 2003a,b, 2004a,b,c) and reference collections from the entomological conservatory at Gembloux Agro-Bio Tech, University of Liege (Department of Functional and Evolutionary Entomology).

### Environmental parameters

As temperature is one of the most important parameters influencing the decomposition rate (Vass et al. 1992; Gill-King 1997; Anderson 2001; Vass 2001; Campobasso et al. 2001; Vass et al. 2002), the ambient air temperature was automatically measured once an hour using a datalogger (Testo 175-T1, www.testo.com) placed on the lateral side of each cage. The daily mean temperature was calculated on the basis of ambient air temperature recorded on a time interval of 24 hours. Other environmental parameters (humidity, wind velocity, wind direction) were recorded thanks to a Vantage Pro Plus™ Stations (Davis instruments, www.davis.com).

### Statistical analyses

Statistical analyses were conducted with R v2.8.0 (www.r-project.org). The number of captured individuals of the two pigs was summed prior to statistical analysis. A generalized linear model with a Poisson error distribution on the absolute count of captured individuals by species and date was first adjusted to confirm the effects of the biotope on the profile of the different species of Silphidae trapped. As the *Thanatophilus* genus was the most abundant genus caught, this model was applied to the *Thanatophilus* genus only. Profiles of the two species of *Thanatophilus* trapped showed similarities within a biotope that were hidden by differences in the total abundance of the species in the sample. That bias was eliminated by fitting a general linear model on the relative abundance of each species, calculated by dividing each insect count by the total abundance of that species for a particular biotope.

## Results

### Environmental parameters

**Figure 2.**
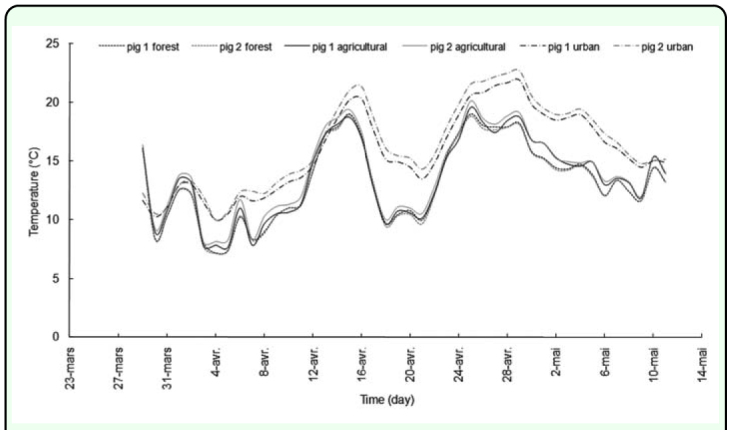
Temperature recordings on the six carcasses (“pig 1 forest” for the first pig carcass on the forest site, “pig 2 forest” for the second carcass on the forest site, “pig 1 agricultural” for the first pig on the agricultural site and “pig 2 agricultural” for the second carcass on the agricultural site, “pig 1 urban” and “pig 2 urban” respectively for the first and the second carcass in the urban site). High quality figures are available online

The mean atmospheric temperature measured during the decompositional process was 13.22° C for the forest site, 13.76° C for the agricultural site and 16.25° C for the urban site (Figure 2). The temperature curves had a similar pattern over time, but the urban site was warmer than the other sites: there was a difference of 3.03° C compared to the forest biotope and 2.49° C compared to the agricultural site. The mean relative humidity was 68.30 % for the “open-air” biotopes and 62.00 % for the urban site.

### Carrion beetles

Seven species of Silphidae were identified during the sampling period, three Nicrophorinae: *Nicrophorus humator* (Gleditsch), *Nicrophorus vespillo* (L.), *Nicrophorus vespilloides* (Herbst) and four Silphinae: *Necrodes littoralis* L., *Oiceoptoma thoracica* L.; *Thanatophilus sinuatus* (Fabricius), *Thanatophilus rugosus* (L.).

**Figure 3.**
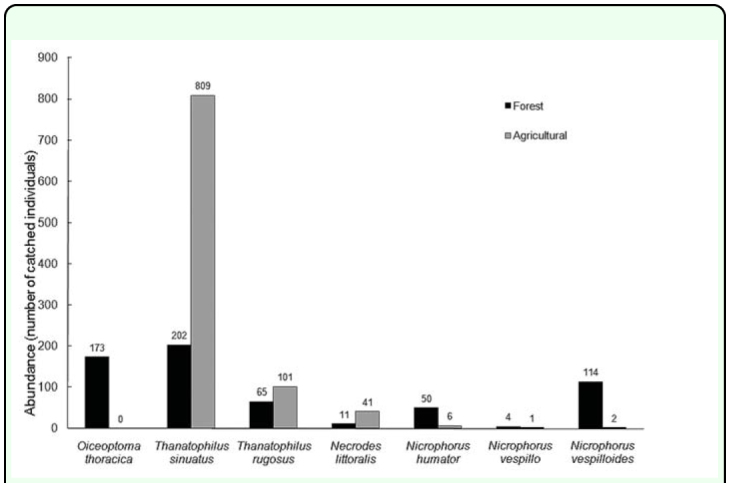
Absolute abundances of Silphidae according to the forest biotope (in dark) and the agricultural biotope (in grey). High quality figures are available online

In total, 1579 individuals were collected at the beginning of spring. Regardless of the biotope, the subfamily of Silphinae (1402 specimens) was more represented than the Nicrophorinae with 177 collected specimens. Silphidae were not found in the urban habitat. Six species of Silphidae were trapped in the agricultural biotope: *N. humator, N. vespillo, N. vespilloides, N. littoralis, T. sinuatus* and *T. rugosus.* In the forest biotope, seven species of Silphidae were collected: *N. humator, N. vespillo, N. vespilloides, N. littoralis, O. thoracica, T. sinuatus* and *T. rugosus.* More individuals were caught in the agricultural biotope (with a total of 960 specimens) than in the forest biotope (619 individuals) (Figure 3).

**Figure 4.**
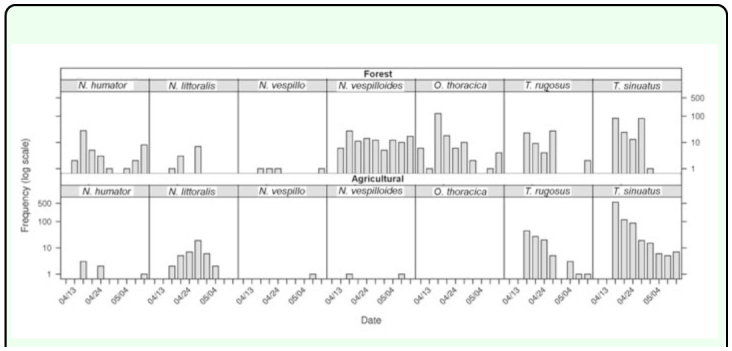
Profiles of Silphidae trapped for the forest and the agricultural biotopes (X-axis represents the time by chronological sampling dates and Y-axis represents the frequency (logarithmic scale). High quality figures are available online

**Figure 5.**
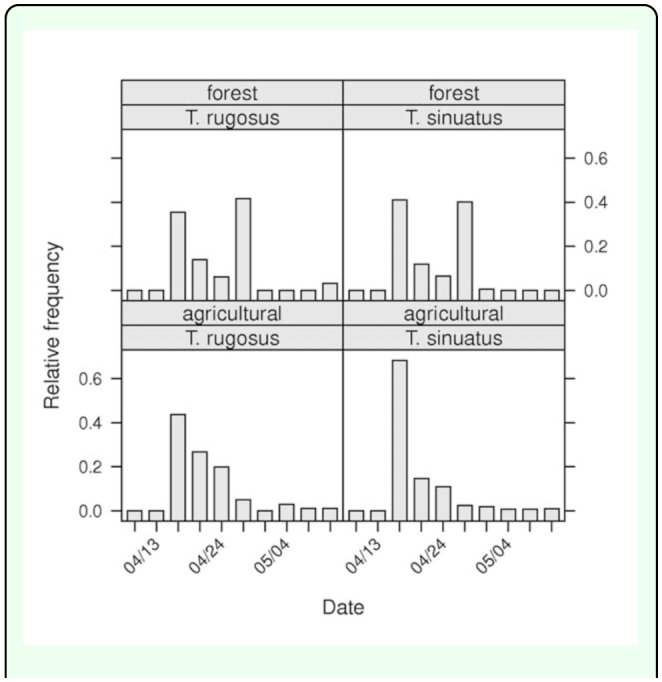
Profiles of members the *Thanatophilus* genus trapped in the forest and the agricultural biotopes. X-axis represents the time by chronological sampling dates and Y-axis represents the relative frequency. High quality figures are available online

Silphinae were more abundant in the agricultural biotope with a total of 951 trapped individuals (60.3% of the total of Silphidae) than in the forest habitat with 451 individuals (28.6%). The Nicrophorinae were more abundant in the forest biotope (168 individuals corresponding to 10.6% of the total of Silphidae) than in the field habitat with only 9 collected individuals (0.6%). The profiles for all species of collected Silphidae are shown in Figure 4. The generalized linear model for all species of Silphidae confirmed significant differences of the profiles between collected species (c^2^_54_ = 451.4, *p* < 0.001) and between habitats (c^2^_9_ = 200.1, *p* < 0.001). In both habitats, *T. sinuatus* was the most abundant species with 809 individuals collected in the agricultural biotope and 202 trapped individuals in the forest habitat. The generalized linear model for the members of the genus *Thanatophilus* trapped confirmed significant differences of the profiles between habitats (c^2^_9_ = 252.5, *p* < 0.001). Despite the similarities observed in Figure 4, the profiles of collections of the two *Thanatophilus* species showed significant differences (c^2^_9_ = 28.6, *p* < 0.001). Since the model is additive and based on absolute counts, this could be a consequence of the significant difference in the total abundance of the two species in the collected sample (c^2^_1_ = 676.1, *p* < 0.001). As expected, the analysis of the relative abundance of the two *Thanatophilus* species (general linear model for the genus *Thanatophilus*) did not show any significant differences between the relative profiles of the two species (F_9,9_ = 2.183, *p* = 0.130). But the relative profiles (Figure 5) of the *Thanatophilus* species trapped were still significantly different between agricultural and forest habitats (F_9_,_9_ = 13.03, *p* < 0.001).

The species *O. thoracica* was exclusively found in the forest habitat (173 individuals). *N. littoralis* (41 specimens) and *T. rugosus* (101 specimens) were more abundant in the agricultural biotope.

Among the Nicrophorinae, *N. vespilloides* was the most abundant species and represented 65.5% of the total of Nicrophorinae, followed by *N. humator* (31.6%) and *N. vespillo* (3.0%).

## Discussion

### Carrion beetles

The absence of Silphidae in the urban biotope can be due to the fact that carcasses were deposited in the second floor of a building in an industrial site. Few forest patches are available for native entomofauna within urban ecosystems (Wolf and Gibbs 2004). The forest fragmentation and urbanization alter the diversity of burying beetles communities (Wolf and Gibbs 2004). Nevertheless, Chauvet et al. (2008) listed Silphidae (*N. humator*) in houses on human cadavers in May in France.

Among the seven species of *Nicrophorus* referenced in Belgium (Hastir and Gaspar, 2001; Ružicka and Schneider, 2004), three of them were observed in early spring: *N. humator, N. vespillo* and *N. vespilloides,* while *N. germanicus, N. investigator, N. interruptus* and *N. vestigator* were not collected. However, these four *Nicrophorus* species are less common and are active later in the season (Hastir and Gaspar 2001; Aleksandrowicz and Komosinski 2005). For example, *N. germanicus* is a rare species and has a very localized distribution (Hastir and Gaspar 2001). Among the Belgian Silphinae, eight species are carrion feeders (necrophagous species) or predators (necrophilous species).

**Table 1.**
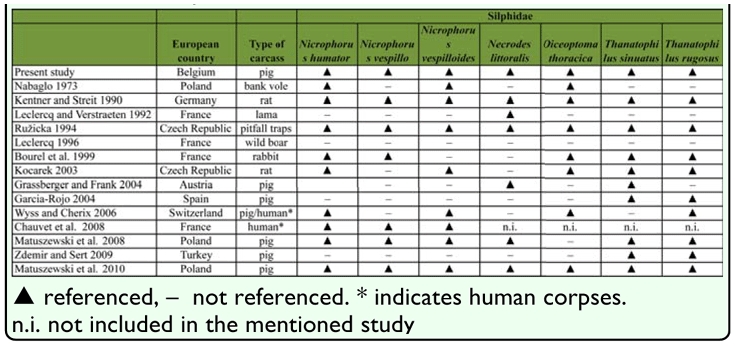
List of the 7 species of Silphidae collected in this study and comparison with literature about insect succession on various types of carcass in Europe

Table 1 compares the species of Silphidae referenced in the literature (in Europe) (Leclercq and Verstraeten 1992; Ružicka 1994; Leclercq 1996; Bourel et al. 1999; Kocarek 2003; Garcia-Rojo 2004; Grassberger and Frank 2004; Wyss and Cherix 2006; Chauvet et al. 2008; Matuszewski et al. 2008; Ozdemir and Sert 2009) and species caught in this study.

In Poland, Matuszewski and colleagues (Matuszewski et al. 2008) found seven species of Silphidae in various forest biotopes (*N. littoralis, T. sinuatus, T. rugosus, N. humator, N. investigator, N. vespilloides* and *N. vespillo*) but they did not collect *O. thoracica* on pig carcasses. However, in further studies of Matuszewski et al. (2010), *O. thoracica* was found exclusively during the spring (from mid-April) in forest habitats (pine-oak, hornbeam-oak and alder forests). The absence of *O. thoracica* in their previous field study (Matuszewski et al. 2008) could be due to the later study time as it was conducted at the end of the summer and the beginning of fall (September 2nd to October 28th) (Matuszewski et al. 2008). Although the literature reports that *O. thoracica* may be found from April to September (Hastir and Gaspar 2001; Debreuil 2004c), field studies suggest that this species has a spring seasonality.

Ružicka (1994) lists 14 species of Silphidae caught in two biotopes (forest and field sites) with pitfall traps in Bohemia. Among the 14 species trapped, seven species are the same as those in the present study but some species that were listed in their study are not carrion feeder such as *Dendroxena quadrimaculata, Aclypea opaca* and *Phosphuga atrata atrata.* These differences in trapping could be due to the fact that some of the pitfall traps were baited with ripened cheese and were attractive for more silphid species than the pig carcasses used in our work. *N. investigator, N. inferruptus* and *N. sepultor* are reported in the Ružicka study (Ružicka 1994); but *N*. *sepultor* does not occur in Belgium (Ružicka and Schneider, 2004). *Silpha tristis* was frequently caught in field sites in central Bohemia (Czech Republic) but not in the present study. This species is not active early in the season. Ružicka reports that the captures are more frequent from June to October (Ružicka 1994; Aleksandrowicz and Komosinski 2005) while Hastir and Gaspar (2001) report a better capture period beginning in May.

Kocarek (2003), collected eight species of necrophagous carrion beetles on exposed rat carcasses and six are the same as in this study: *N. humator, N. vespilloides, N. vespillo, T. rugosus, T. sinuatus* and *O. thoracica.* The two species additionally found in Kocarek study were *N. interruptus* and *N. investigator,* but these species are not found in early spring. *N. interruptus* shows a seasonal preference for summer (July–August), whereas *N. investigator* for summer and fall (Kocarek 2003; Aleksandrowicz and Komosinski 2005).

In their study on decaying rodent carcasses, Kentner and Streit (1990) report five Nicrophorinae (*N. interruptus, N. humator, N. investigator, N. vespillo, N. vespilloides*) but *N. interruptus* and *N. investigator* were collected in small numbers (Kentner and Streit 1990). *N. vespilloides* was the dominant species in the forest habitat and was not collected in the open field site, while *N. vespillo* and *N. humator* were collected in bothopen field and forest. However, *N. vespillo* showed a preference for the open field habitat and *N. humator* for the forest habitat. Six Silphinae were reported (Kentner and Streit 1990): *N. littoralis, O. thoracica, S. obscura, S. tristis, T. sinuatus, T. rugosus.* Any *Thanatophilus* spp. and *Silpha* spp. were collected in a forest biotope, while *N. littoralis* and *O. thoracica* were exclusively found in an open field biotope. The dominant species of Silphinae was *T. sinuatus* (Kentner and Streit 1990). An entomofaunal colonization study on rabbit carcasses in sand dune lists five species of Silphidae in the spring: *N. humator, N. vespillo, O. thoracica, T. sinuatus* and *T. rugosus. T. sinuatus* was the most abundant species trapped (Bourel et al. 1999).

As *O. thoracica* is a forest inhabiting species and its absence in the agricultural biotope in our study is not surprising. Other studies (Kentner and Streit 1990; Kocarek 2001; Kocarek 2003) have shown the forest preference of *O. thoracica.* However, *O. thoracica* has been reported in open biotope in Northern France (sand dune) (Bourel et al. 1999). *N. vespilloides* and *N*. *humator* are also considered to be forest species (Kentner and Streit 1990; Scott 1998; Hastir and Gaspar 2001; Kocarek 2001; Kocarek 2003; Aleksandrowicz and Komosinski 2005). This ecological habitat preference explains the great difference we observed between both biotopes with clearly more trappings for the forest biotope. *Thanatophilus* spp. are referenced as field or meadow species (Ružicka 1994; Hastir and Gaspar 2001; Kocarek 2001; Kocarek 2003; Aleksandrowicz and Komosinski 2005). The latter species were more abundant in the agricultural biotope than in the forest habitat in our study. Ružicka (1994) and Kocarek (2001; 2003) show the same habitat preference: *T. sinuatus* (Ružicka 1994; Kocarek 2003) and *T. rugosus* (Kocarek 2003) were collected mostly in the field sites. However, Matuszewski and colleagues (Matuszewski et al. 2008; Matuszewski et al. 2010) have collected numerous adults of *Thanatophilus* spp. on pig carcasses located in various forest habitats. As in our work, *T. sinuatus* was found early in the season starting in April by Ružicka (1994). In a suburban area of Madrid (Spain) (Garcia-Rojo 2004), *T.* *rugosus* and *T. sinuatus* were also found from mid-April on decaying pigs.

*N. vespillo,* a meadow species (Scott 1998; Kentner and Streit 1990), has been less trapped during the sampling period (early spring) and the comparison between the two biotopes is not relevant. The small number of collections ,may be due to the fact that *N. vespillo* becomes reproductively active in summer (May–July) (Scott 1998), while other species of *Nicrophorus* have a reproductive period in spring starting in April (Scott 1998) or later (*N. interruptus, N. investigator*) (Kentner and Streit 1990). *N. littoralis* has been collected in both biotopes. However, several studies (Kentner and Streit 1990; Ružicka 1994) report that *N. littoralis* was absent in open field habitats and only collected in forest habitats. It was a dominant species of carrion beetles (larvae and adults) collected in forest studies of Matuszewski and colleagues (Matuszewski et al. 2008; Matuszewski et al. 2010). Concerning Silphinae, they are active from April to September (Hastir and Gaspar 2001; Peck 2001). However, some species of Silphinae seem to have seasonal preferences (e.g., *O. thoracica* in spring).

Food source preferences (niche differentiation) and carcass size could explain the great differences observed between the two subfamilies (Scott 1998; Watson and Carlton 2005; Ikeda et al. 2006). Nicrophorinae (*Nicrophorus* spp.) prefer small vertebrate carcasses (rodents, birds) because of their burying behaivor, and their subsocial behaviour for breeding their offspring (biparental care) (Pukowski 1933; Anderson 1982; Kentner and Streit 1990; Trumbo 1992; Scott 1998; Ikeda et al. 2006; Sikes 2008). However, reproductively immature *Nicrophorus* could be found on larger carcasses (> 300g) where they feed on fly eggs or maggots (necrophilous species) and rarely on decaying meat (Kentner and Streit 1990; Scott 1998; Matuszewski et al. 2008; Sikes 2005; Sikes 2008). Contrary to Nicrophorinae, Silphinae show no parental care (Ratcliffe, 1996). Silphine species tend to use larger carcasses for reproduction and larval development (Ratcliffe 1996; Bishop 2001; Hoback et al. 2004). Pig carcasses are considered as large carcasses and suitable for Silphinae.

Concerning the decompositional stage preference, Silphidae tend to arrive during the mid-stage of decay (Ratcliffe 1972; Ratcliffe 1996; Hoback et al. 2004). Other studies (Kocarek 2003; Matuszewski et al. 2008; Matuszewski et al. 2010) associate silphid activity of *N. littoralis, Thanatophilus* spp. and *Nicrophorus* spp. on carcasses during the active decay stage. However, an African species of Silphidae, *Thanatophilus micans,* could be found within 24 hours of death on animal carcasses (Midgley et al. 2009).

In carrion beetles communities, niche differentiation can occur along dimensions of season, habitat (biotope) and carcass size (Scott 1998; Kentner and Streit 1990; Kocarek 2001; Hocking et al. 2007).

Further studies on carrion beetles (Coleoptera: Silphidae) and insect postmortem colonization are currently being conducted at the Department of Functional and Evolutionary Entomology (Belgium, Gembloux Agro-Bio Tech, University of Liege).

### Self-critique

In this experiment, an infrequent sampling (every fourth days) was used for collecting sarcosaprophagous insects in a six-week period on decaying pigs. The use of soapy water in traps without any preservative solution is not recommended for infrequent sampling. Indeed, insects themselves could rot in pitfall and yellow traps, although this is less of a problem for adults. For further field studies, it would be better to use traps filled with ethylene glycol (50%) in case of infrequent sampling or use a daily sampling. Only adult stages were included in this study and there is no information about immature stages of carrion beetles. However, for forensic purposes, it is important to have information about the presence/absence of these immature stages.
